# *Chlamydia pneumoniae* Infection Induces Vascular Smooth Muscle Cell Migration and Atherosclerosis Through Mitochondrial Reactive Oxygen Species-Mediated JunB-Fra-1 Activation

**DOI:** 10.3389/fcell.2022.879023

**Published:** 2022-04-12

**Authors:** Xi Zhao, Guolin Miao, Lijun Zhang, Yuke Zhang, Huanhuan Zhao, Zhelong Xu, Beibei Wang, Lijun Zhang

**Affiliations:** ^1^ Department of Physiology and Pathophysiology, School of Basic Medical Sciences, Tianjin Medical University, Tianjin, China; ^2^ State Key Laboratory of Natural and Biomimetic Drugs, School of Pharmaceutical Sciences, Peking University, Beijing, China; ^3^ Institute of Cardiovascular Sciences and Key Laboratory of Molecular Cardiovascular Sciences, Ministry of Education, Peking University, Beijing, China

**Keywords:** atheroclerosis, *Chlamydia pneumloniae*, vascular smooth muscle cell, Activator protein 1, mitochondrial reactive oxygen species

## Abstract

Infection is closely related to atherosclerosis, which is a major pathological basis for cardiovascular diseases. Vascular smooth muscle cell (VSMC) migration is an important trigger in development of atherosclerosis that is associated with *Chlamydia pneumoniae* (*C. pneumoniae*) infection. However, the mechanism of VSMC migration remains unclear, and whether antioxidant could be a therapeutic target for *C. pneumoniae* infection-induced atherosclerosis also remains unknown. The results showed that *C. pneumoniae* infection mainly impaired mitochondrial function and increased the level of mitochondrial reactive oxygen species (mtROS). The expressions of protein JunB, Fra-1 and Matrix metalloproteinase 2 (MMP) evidently increased after *C. pneumoniae* infection, and the interaction between JunB and Fra-1 was also enhanced. After scavenging mtROS by antioxidant Mito-TEMPO, the increasing expressions of JunB, Fra-1, MMP2 and the capacity of VSMC migration induced by *C. pneumoniae* infection were all inhibited. In comparison with infected ApoE^-/-^ mice, the level of ROS in atherosclerotic lesion in ApoE^-/-^TLR2^-/-^ mice with *C. pneumoniae* infection decreased. Knocking out TLR2 suppressed the expressions of JunB, Fra-1 and MMP2 in VSMCs and the formation of atherosclerotic lesion after *C. pneumoniae* infection. Furthermore, after using small interfering RNA to inhibit the expression of TLR2, the level of mtROS and the expressions of JunB, Fra-1 and MMP2 apparently decreased. Taken together, *C. pneumoniae* infection may promote VSMC migration and atherosclerosis development by increasing the level of mtROS through TLR2 to activate the JunB-Fra-1/MMP2 signaling pathway. The data provide the first evidence that antioxidant could reduce *C. pneumoniae* infection-induced VSMC migration and atherosclerosis.

## Introduction

Cardiovascular disease is a leading cause of morbidity and mortality in developed countries, and its prevalence is increasing at an alarming rate in developing countries (Virani et al., 2020). Atherosclerosis is the major pathological basis for cardiovascular diseases. Despite the advance of therapies to lower lipids and reduce hypertension, atherosclerosis is still a plague to society (Brophy et al., 2019). Thus, understanding the molecular mechanisms involved in atherogenesis is critical for developing novel therapies for this devastating disease. Vascular smooth muscle cells (VSMCs) are main constitutive stromal cells of the vascular wall, assuming a variety of different structural and physiological functions (Bennett et al., 2016). VSMCs switched from a quiescent contractile phenotype to a synthetic phenotype could migrate from the media into the intima and proliferate there, and have additive effects on atherosclerotic lesion formation (Chappell et al., 2016). Therefore, clarifying the mechanisms of VSMC migration will help to develop the therapeutic interventions for aberrant VSMC migration-related diseases (e.g. atherosclerosis).

Over the past decades, emerging evidence has been reported that atherosclerosis is a chronic inflammatory disease, suggesting that chronic infection has a role in the development of atherosclerosis (Pothineni et al., 2017; Li et al., 2020b). Intracellular pathogens *Chlamydia pneumoniae* (*C. pneumoniae*) can be transferred from the lungs to the vessel wall, and then infect VSMCs (Gaydos et al., 1996). This infection is often undetected, and thus persists (Roufaiel et al., 2016). Our previous research showed that *C. pneumoniae* infection induced aberrant VSMC migration, thereby promoting atherosclerosis in ApoE^‐/‐^ mice (Miao et al., 2020). But VSMC migration is a very complicated process, and is finely regulated by multiple factors. Therefore, the mechanisms of VSMC migration require further elucidation.

It has been reported that mitochondrial dysfunction could induce a series of pathophysiological processes including cell migration (Gaude et al., 2018), which is closely related to atherosclerotic lesion formation (Zhu et al., 2019; Miao et al., 2020). And increased mitochondrial reactive oxygen species (mtROS) are major signs of mitochondrial dysfunction (Wang et al., 2020). Chen et al. found that astaxanthin could mitigate hypertensive vascular remodeling by decreasing the overproduction of mtROS and VSMC migration, indicating that mtROS plays an important role in VSMC migration (Chen et al., 2020). But the therapeutic effects of antioxidant therapy for *C. pneumoniae* infection-induced VSMC migration and atherosclerosis still need to evaluate.

Activator protein 1 (AP-1), a dimeric transcription factor, has been reported to regulate a number of targets involved in cell survival, apoptosis and migration in a context-dependent manner (Shaulian and Karin, 2002). Previous studies showed that JunB Proto-Oncogene (JunB) and FBJ osteosarcoma oncogene related antigen 1 (Fra-1) were the main contributors to AP-1 binding activity in VSMCs (Farzaneh-Far et al., 2001). The activation of JunB could induce the migration of VSMCs and retinal microvascular endothelial cells (Li et al., 2013; Kumar et al., 2020). In addition, migration capacity of VSMCs (Cao et al., 2006), esophageal squamous cells (Chunni Wang et al., 2017) and mammary epithelial cells (Bakiri et al., 2015) is governed by Fra-1. Furthermore, primary human coronary artery endothelial cells infected with *C. pneumoniae* could increase the expression of JunB (Wang et al., 2013). Hence it is possible that *C. pneumoniae* infection induces VSMC migration through activating JunB-Fra-1 heterodimer.

Accordingly, we demonstrated that *C. pneumoniae* infection increased the level of mtROS in VSMCs due to mitochondrial dysfunction in the present study. mtROS accumulation activated JunB-Fra-1-mediated signal pathway to promote *C. pneumoniae* infection-induced VSMC migration and atherosclerotic lesion formation.

## Materials and Methods

### Animals

ApoE^‐/‐^ (C57BL/6-Apoe^em1Cd82/Nju^) mice and C57BL/6J, TLR2^‐/‐^ (B6/JGpt-Tlr2^em1Cd/Gpt^) mice were purchased from GemPharmatech Co., Ltd. (Jiangsu, China). The ApoE^‐/‐^ mice were crossed with TLR2^‐/‐^ mice to generate TLR2^‐/‐^ApoE^‐/‐^ mice. All mice were housed in a facility with a 12 h light/12 h dark cycle and given free access to water and standard rodent chow. All animal protocols conformed to the Guidelines for the Care and Use of Laboratory Animals, and were prepared and approved by the Animal Care and Use Committee of Tianjin Medical University (Approval No. TMUaMEC2018007). Mice were fed a Western diet with regular casein, 1.25% added cholesterol and 0.5% sodium cholate (D12109C; Research Diets, Brunswick, NJ, United States) at 8 weeks old, and were sacrificed after 6 weeks. Infected mice received 40 μl sucrose-phosphate-glutamate (SPG) buffer (pH 7.2) containing 2 × 10^7^ inclusion forming units (IFU) of gradient-purified *C. pneumoniae* strain AR39 (#53592, ATCC, Virginia, United States) every 2 weeks for 6 weeks, and mock-infected mice received 40 μl SPG alone without *C. pneumoniae*.

### Histological Analysis

Hearts were harvested and embedded in Optimal Cutting Temperature (O.C.T.) compound (#4583, Sakura Finetek, California, United States) for cryo-sectioning. Serial sections were cut at 7 μM thickness and every microscope slide had nine sections.

Dihydroethidium (DHE) (BB47051, BestBio, Beijing, China) was used to analyze the level of cellular ROS in aortic sinus. Briefly, sections were washed with phosphate buffered saline (PBS) (A19711, Chuanqiu Biotechnoloyg Co., Ltd., Shanghai, China), and covered with staining working solution for 30 min. Thereafter, samples were observed with a laser scanning confocal microscope.

For immunofluorescence staining, after washing twice with PBS, sections were incubated with 5% nonfat milk (N7861, LABLEAD Inc., Beijing, China) for 1 h. Sections were then incubated overnight at 4°C with primary antibodies against α-SMA (#48938S, mouse, 1:75, Cell Signaling, Massachusetts, United States), JunB (#3753S, rabbit, 1:75, Cell Signaling, Massachusetts, United States), Fra-1 (sc-183, rabbit, 1:20, Santa Cruz Biotechnology, Texas, United States), and Matrix metalloproteinase 2 (MMP) (ab92536, rabbit, 1:75, Abcam, Cambridge, United Kingdom). The next day, sections were washed, and incubated with the appropriate secondary antibody Alexa Fluor^®^488 Conjugate anti-mouse IgG (#4408, goat, 1:200, Cell Signaling, Massachusetts, United States), or Alexa Fluor^®^594 Conjugate anti-rabbit IgG (#77344, goat, 1:200, Cell Signaling, Massachusetts, United States) for 60 min at room temperature. Blinded image analysis was performed by Image Pro Plus 6.0 software.

### Cell Culture and Small Interfering RNA (siRNA) Transfection

Rat primary thoracic aortic VSMCs were cultured in DMEM supplemented with 10% FBS and maintained at 37°C in a 5% CO_2_ atmosphere. siRNAs targeting TLR2, Fra-1, JunB, MMP2 and scramble siRNAs (scrRNA) in the rat transcriptome were generated by Thermo Fisher Scientific (Massachusetts, United States). For transfection, cells were seeded in 6-well plate. When cell confluency was 60–80%, RNA-lipid complexes (#13778030, Thermo Fisher Scientific) were added and then the cells were incubated for 48 h to decrease the expressions of targeting proteins.

### Western Blot

Cells were lysed in RIPA lysis buffer (P0013B, Beyotime Biotechnology, Beijing, China) containing protease-phosphatase cocktail inhibitor mix (#87786, Thermo Fisher Scientific, Massachusetts, United States) for 30 min at 4°C and harvested by a cell scraper. Protein concentrations were determined using a BCA Protein Assay Kit (PC0020, Solarbio, Beijing, China). Cells were incubated in primary antibodies against TLR2 (ab209217, rabbit, 1:1000, Abcam, Cambridge, United Kingdom), JunB (3753S, rabbit, 1:1000, Cell Signaling, Massachusetts, United States), Fra-1 (sc-28310, mouse, 1:200, Santa Cruz Biotechnology, Texas, United States), MMP2 (ab92536, rabbit, 1:1000, Abcam, Cambridge, United Kingdom), and β-actin (3700, mouse, 1:5000, Cell Signaling, Massachusetts, United States) overnight at 4°C. After incubation with the corresponding HRP-conjugated secondary antibodies (#7076, mouse, 1:2000; #7074, rabbit, 1:2000, Cell Signaling, Massachusetts, United States) for 2 h at room temperature, the blots were detected with an enhanced Chemiluminescence Kit (P0018FM, Beyotime Biotechnology, Beijing, China).

### Co-Immunoprecipitation and Immunoblot Analysis

500 μg cellular protein, 2 μg antibody, and 20 μl Protein A/G PLUS-Agarose (sc-2003, Santa Cruz Biotechnology, Texas, United States) were mixed with rocking at 4°C overnight. The following antibodies were used: JunB (3753S, Cell Signaling, Massachusetts, United States), Fra-1 (sc-28310, Santa Cruz Biotechnology, Texas, United States) and IgG (#3420S, Cell Signaling, Massachusetts, United States). The next day, Protein A/G PLUS-Agarose was centrifuged for 5 min at 1,000 × g to collect for immunoblot. Protein A/G PLUS-Agarose was resuspended in 2 × SDS-PAGE loading buffer (P1019, Solabio, Beijing, China), and then performed Western blot.

### Transwell Assay

Cell migration was assessed using a modified Boyden’s chamber method. Cells were harvested after various treatments in different groups. Cells were seeded at a density of 1.5 × 10^4^ cells/well in a 24-well plate. Medium containing 30% FBS was used to induce cell migration. Cells were allowed to migrate for 8 h, and then stained with crystal violet and counted under a phase contrast microscopy.

### Wound Healing Assay

VSMCs were seeded in the 6-well plate and cultured until cell monolayers were formed. Monolayers were wounded by manual scraping with a sterile 20–200 μl pipette tip, and then cell debris was washed with PBS. Images were acquired immediately following complete media replacement (T0). After 24 h, wound pictures were acquired again (T24) and assessed using Image Pro Plus 6.0 software. Briefly, extents of closure at T24 were calculated by subtracting area at T0. The percentage closure was determined by normalizing difference to area at T0. Each experiment was performed four times using triplicate wells.

### JC-1 Staining

Mitochondrial Membrane Potential Assay Kit (JC-1) was bought from Beyotime (C2006, Beijing, China). Treated cells were incubated with JC-1 staining working solution in serum-free DMEM medium for 20 min at 37°C in dark. After washing twice, mitochondrial membrane potential assay was carried out using a confocal microscope.

### Quantitative Real-Time PCR (qPCR)

mtDNA copy number was analyzed by qPCR. Total RNA was extracted from VSMCs using TRIzol (#15596026, Thermo Scientific, Massachusetts, United States), and first-strand cDNA was prepared using the TransScript RT enzyme (AT411-02, TransGen Biotech, Beijing, China). qPCR was performed using 1,000 ng DNA as the starting material with the designed primers for the mitochondrial gene cytochrome oxidase subunit 1 (Forward: ATT​GCC​CTC​CCC​TCT​CTA​CGC​A; Reverse: CGT​AGC​TTC​AGT​ATC​ATT​GGT​GCC​C) and nuclear DNA products *β*-actin (Forward: CCA​TGT​TCC​AAA​ACC​ATT​CC; Reverse: GGG​CAA​CCT​TCC​CAA​TAA​AT). Relative values of mitochondrial DNA products (COX-1) and nuclear DNA products (β-actin) in each sample were used.

### Intracellular Adenosine Triphosphate (ATP) Measurement

ATP was detected by ATP Assay Kit (S0026, Beyotime, Beijing, China) following the manufacturer’s protocol. Briefly, cells were gathered by centrifuging for 5 min at 1,500 rpm and lysed in the lysis solution (AP01L013, Life-iLab, Shanghai, China). Supernatant was collected after centrifuging for 5 min at 12,000 × g and then diluted to one 10th with ATP detection reagent dilution. 100 μl ATP detection working solution were mixed with 20 μl prepared standards or samples in 96-well plate. The results were detected by a luminometer (Synergy NEO, BioTek Instruments).

### Cellular ROS Measurement

Cells were seeded onto 96-well plate (3,000 cells/well) and allowed to adhere overnight. After treating cells, culture medium was removed, and cells were incubated with CM-H2DCFDA (10 μM, S0033, Beyotime, Beijing, China) in serum-free DMEM for 20 min at 37°C. Harvested cells were washed twice with PBS and resuspended in 200 µl PBS. Cellular ROS levels were measured using fluorescent microplate reader analysis.

### mtROS Measurement

Mito-TEMPO (T19428, Targetmol, MA, United States) was used to eliminate mtROS in VSMC. VSMCs plated on glass bottom dishes were treated as indicated, and then were incubated with a 5 μM MitoSOX™ reagent working solution (M36008, Invitrogen, Massachusetts, United States) and 20 nM Mito-tracker green (C1048, Beyotime, Beijing, China) in dark for 10 min at 37°C. mtROS level was measured by a laser scanning confocal microscope.

### Quantitative Proteomic Analysis by Tandem Mass Tag Technology

The treated cells were lysed and centrifugated at 14,000 × g for 30 min, the supernatant was quantified with the BCA Protein Assay Kit (PC0020, Solarbio, Beijing, China). 100 μg of the protein from each sample was labeled using Tandem Mass Tag™ 6-plex (TMTsixplex™) according to the manufacturer’s instructions (90064CH, Thermo Fisher Scientific, Massachusetts, United States). After TMT labeling, the labeled digest samples were fractionated, and 10 fractions were obtained. The surplus labels and salts were diminished using The High pH Reversed-phase Peptide Fractionation Kit (#84868, Thermo Fisher Scientific, Massachusetts, United States). Liquid chromatography–mass spectrometry/mass spectrometry (LC-MS/MS) analysis was performed on a Q Exactive mass spectrometer (Thermo Fisher Scientific, Massachusetts, United States) that was coupled to Easy nLC (Proxeon Biosystems, Thermo Fisher Scientific, Massachusetts, United States of America) for 60 min. The MS raw data for each sample were searched using the MASCOT engine (Matrix Science, London, United Kingdom; version 2.2) embedded into Proteome Discoverer 1.4 software for identification and quantitation analysis. The differentially expressed proteins were identified by fold change values, and the fold change was set as > 1.2 or <0.8. A *p* value (Student’s *t*-test) of <0.05 was considered statistically significant.

### Statistical Analysis

Statistical analysis was performed with GraphPad Prism 8.3 (GraphPad Software, San Diego, CA). Data are presented as means ± standard error of mean (SEM). Statistical significance (*p* values) was calculated using Student’s *t*-tests or one-way analysis of variance (ANOVA). *p* < 0.05 was considered statistically significant. No statistical methods were used to predetermine sample size.

## Results

### *C. pneumoniae* Infection Causing Mitochondrial Dysfunction and Increased mtROS are Necessary for VSMC Migration

**FIGURE 1:**
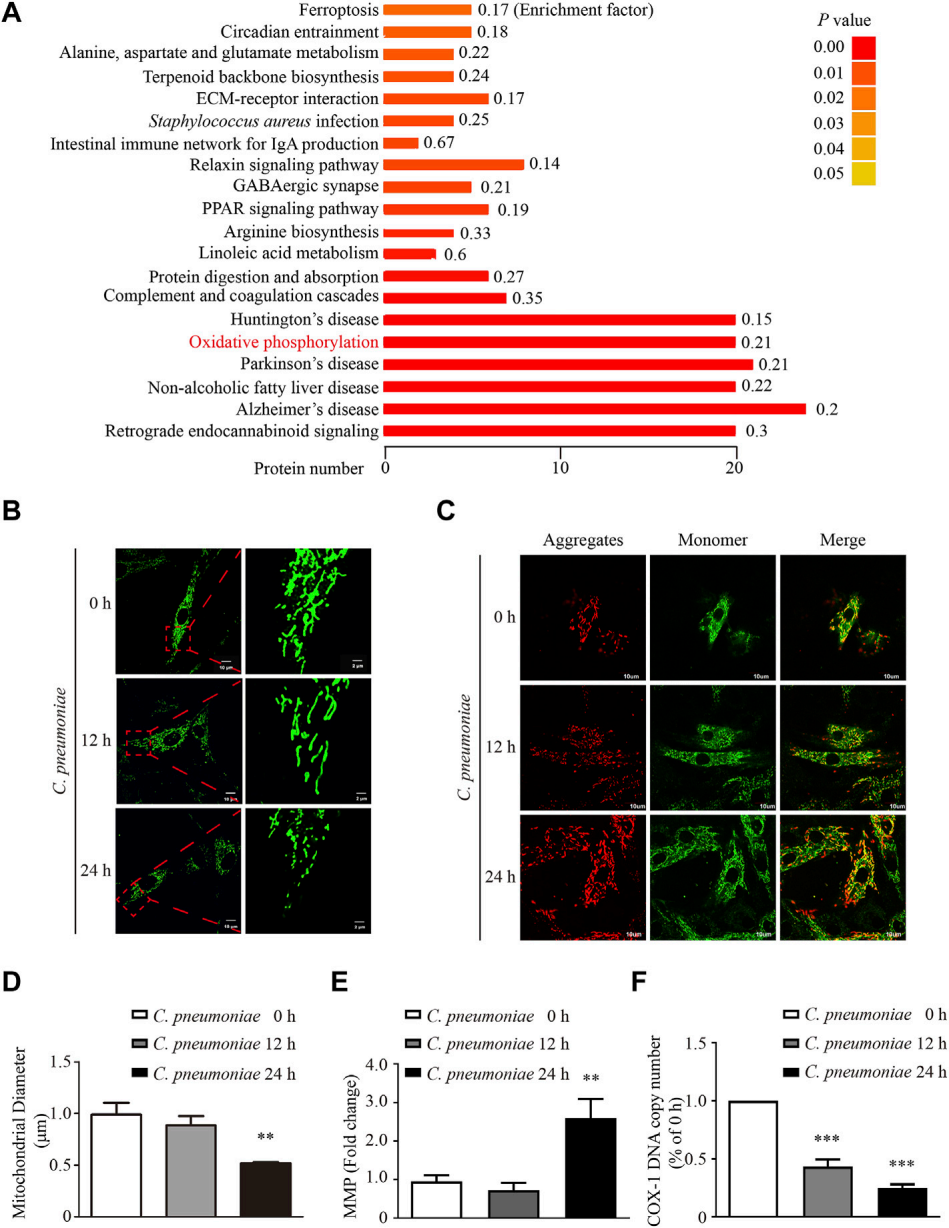
*C. pneumoniae* infection induces mitochondrial dysfunction in VSMCs. VSMCs were infected with *C. pneumoniae* (5 × 10^5^ IFU). **(A)** Differentially expressed proteins in KEGG pathway in VSMCs after *C. pneumoniae* infection. **(B)** Mito-Tracker Green staining showed mitochondrial morphology in VSMCs. Scale bar: 10 μM (Left), 2 μM (Right). **(C)** Mitochondrial membrane potential (MMP) is indicated by JC-1. Scale bar: 10 μM. **(D)** Quantitative analyses of mitochondrial length in VSMCs. **p < 0.01, compared with *C. pneumoniae* infection 0 h group, as analyzed by One-Way ANOVA. **(E)** Quantitative analyses of mitochondrial membrane potential (MMP). JC-1 monomer yields green fluorescence due to low mitochondrial membrane potential; JC-1 aggregates yielding a red fluorescence due to high mitochondrial membrane potential. **p < 0.01, compared with *C. pneumoniae* infection 0 h group, as analyzed by Student’s *t*-test. **(F)** COX-1 DNA copy number in different groups. ***p < 0.001, compared with *C. pneumoniae* infection 0 h group, as analyzed by One-Way ANOVA.

**FIGURE 2:**
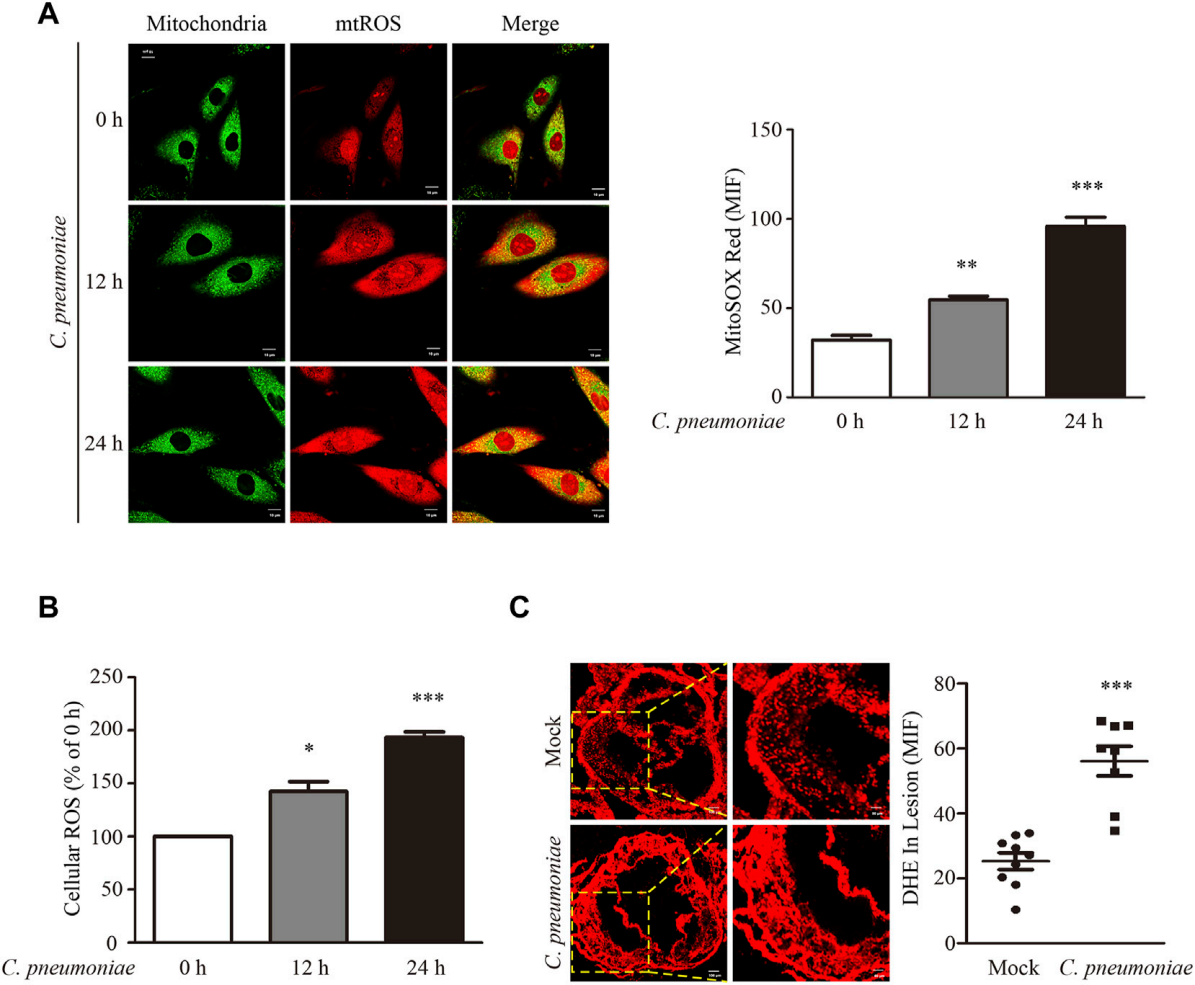
*C. pneumoniae* infection increases the level of mtROS in VSMCs. **(A,B)** VSMCs were infected with *C. pneumoniae* (5 × 10^5^ IFU) for 0, 12, and 24 h. **(A)** Representative confocal microscopy images of VSMCs that were stained with MitoSOX Red and Mito-Tracker Green and quantification of mean fluorescence intensity in VSMCs after *C. pneumoniae* infection. Scale bar: 10 μM **p < 0.01, ***p < 0.001, compared with *C. pneumoniae* infection 0 h, as analyzed by one-way ANOVA. MIF, mean intensity of fluorescence. **(B)** Quantification of the level of cellular ROS in VSMCs infected with *C. pneumoniae* for the indicated timepoints. *p < 0.05, ***p < 0.001, compared with *C. pneumoniae* infection 0 h, as analyzed by one-way ANOVA. **(C)** ApoE^‐/‐^ mice (*n* = 9) were infected intranasally with 40 μl sucrose-phosphate-glutamate (SPG) buffer (pH 7.2) containing *C. pneumoniae* (2 × 10^7^ IFU). For the mock infection, ApoE^‐/‐^ mice (*n* = 8) were intranasally administrated with SPG. All mice were analyzed after 6 weeks of Western diet. ROS level was assessed by Dihydroethidium (DHE) in the lesions of aortic sinus. Scale bar: 100 μM (Left), 50 μM (Right). ***p < 0.001, compared with mock infection, as analyzed by Student’s *t*-test.

**FIGURE 3:**
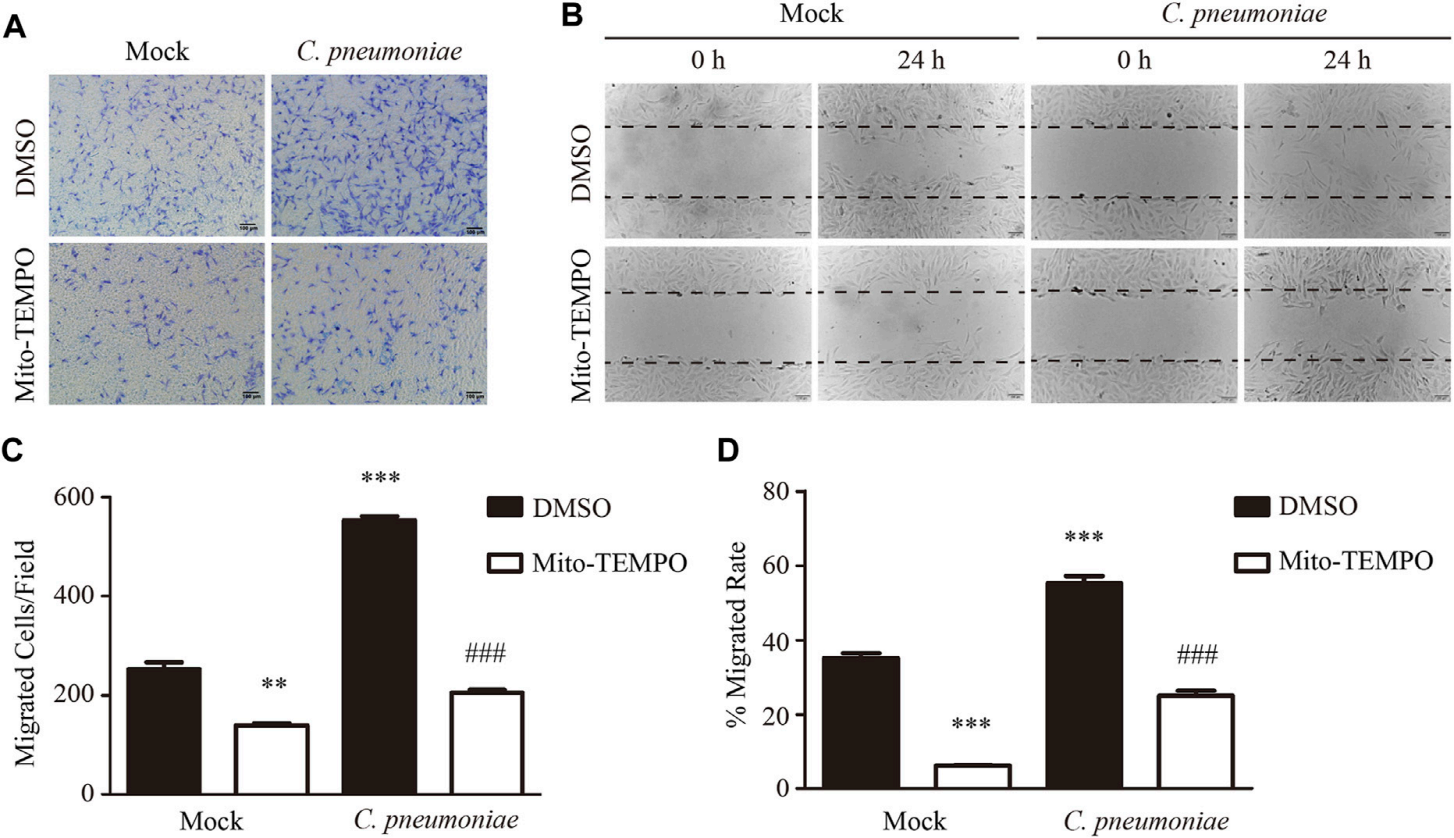
*C. pneumoniae* infection induces VSMC migration through mtROS. **(A−D)** VSMCs were infected with *C. pneumoniae* (5 × 10^5^ IFU). Mito-TEMPO (7 μM, 30 min prior to *C. pneumoniae* infection) eliminated mtROS and inhibited *C. pneumoniae* infection-induced VSMC migration. **(A)** Representative images of VSMC migration in Transwell assays. Scale bar: 100 μM. **(B)** Representative images of VSMC migration in wound healing assays. Scale bar: 100 μM. **(C)** The quantitative results for migrated VSMCs/field. **p < 0.01, ***p < 0.001, compared with mock infection and DMSO group, as analyzed by Student’s *t*-test. ^###^ *p* < 0.001, compared with mock infection and Mito-TEMPO group, as analyzed by Student’s *t*-test. **(D)** The quantitative results for the area of wound closure were detected at 24 h after scratch. ***p < 0.001, compared with mock infection and DMSO group, as analyzed by Student’s *t*-test. ^###^ *p* < 0.001, compared with mock infection and Mito-TEMPO group, as analyzed by Student’s *t*-test.

To study the impact of *C. pneumoniae* infection on VSMC functions, we performed quantitative proteomic analysis by using Tandem mass tag (TMT) technology and Kyoto Encyclopedia of Genes and Genome (KEGG) enrichment analysis, and found that oxidative phosphorylation (OXPHOS)-related expression signature from VSMCs was enriched in response to *C. pneumoniae* infection (Figure 1A, Supplementary Table). Mitochondrial primary function is to produce energy in the form of ATP through OXPHOS. Therefore, we speculated that *C. pneumoniae* infection may affect mitochondrial function in VSMCs. We examined the effect of *C. pneumoniae* infection on mitochondrial functional parameters in VSMCs, and found that the copy number of mitochondria decreased (Figure 1F), the length of mitochondria markedly shortened (Figures 1B,D) and mitochondrial membrane potential significantly increased (Figures 1C,E) after *C. pneumoniae* infection. These results suggest that *C. pneumoniae* infection could lead to mitochondrial dysfunction in the infected VSMCs. In eukaryotes, the vast majority of cellular ROS (approximately 90%) can be traced back to the mitochondria (Balaban et al., 2005). To further explore the role of *C. pneumoniae* infection in mitochondrial dysfunction, we tested the levels of mtROS and cellular ROS in VSMCs and atherosclerotic lesion. Excitingly, the levels of mtROS and ROS were both higher than that in the uninfected VSMCs (Figures 2A,B). Moreover, ROS levels in the lesions of aortic sinus in the infected ApoE^‐/‐^ mice also increased by approximately 121.84% compared with mock infected ApoE^‐/‐^ mice (Figure 2C). mtROS is closely related to cell migration (Chen et al., 2020). Hence, we investigated whether the level of mtROS impacts VSMC migration. We used 7 µM Mito-TEMPO to eliminate mtROS in VSMC, which is the highest-efficacy with the lowest-toxicity (Supplementary Figure) and found that VSMC migration induced by *C. pneumoniae* infection was significantly inhibited by Mito-TEMPO in both Transwell (Figures 3A,C) and wound healing assays (Figures 3B,D).

### JunB/Fra-1/MMP2 Mediates *C. pneumoniae* Infection-Induced VSMC Migration

**FIGURE 4:**
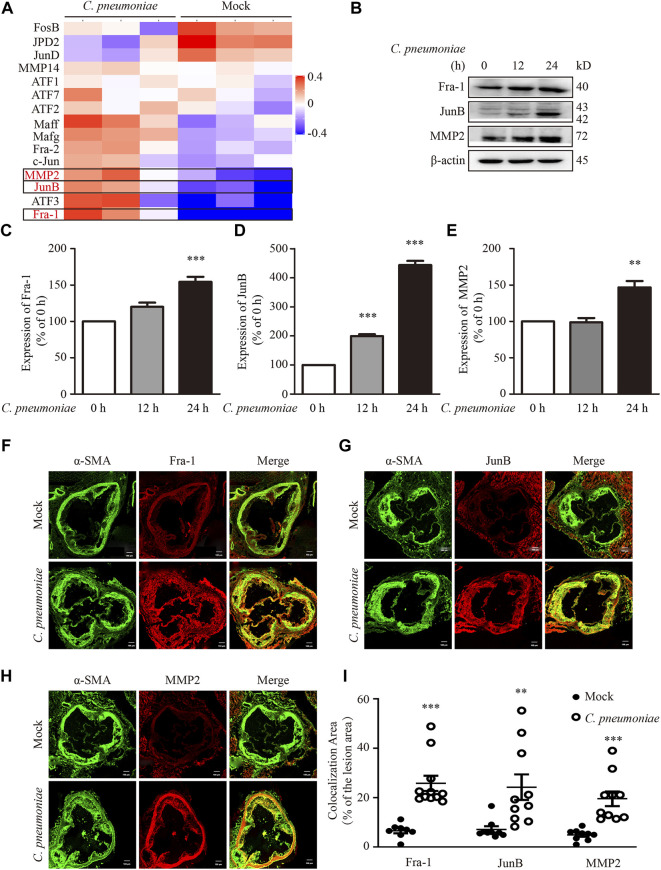
*C. pneumoniae* infection upregulates Fra-1, JunB and MMP2 expressions. **(A,B)** VSMCs were infected with *C. pneumoniae* (5 × 10^5^ IFU). **(A)** Differentially expressed proteins in AP-1 family and MMP family members in VSMCs after *C. pneumoniae* infection. **(B)** Representative Western blot results of Fra-1, JunB and MMP2 from VSMCs that had been infected with *C. pneumoniae* for the indicated timepoints. **(C–E)** The expression ratio of the indicated protein to β-actin from three independent experiments is presented. **p < 0.01, ***p < 0.001, compared with *C. pneumoniae* infection 0 h group, as analyzed by one-way ANOVA. **(F–H)** The expressions of Fra-1 **(F)** or JunB **(G)** or MMP2 **(H)** in VSMCs in atherosclerotic lesions of ApoE^‐/‐^ mice fed a Western diet for 6 weeks with or without *C. pneumoniae* infection. Scale bar: 100 μM. **(I)** Quantification of active Fra-1^+^, JunB^+^ and MMP2^+^ VSMCs in the lesions of aortic sinus from ApoE^‐/‐^ mice fed a Western diet for 6 weeks with or without *C. pneumoniae* infection. **p < 0.01, ***p < 0.001, compared with mock infection groups, as analyzed by Student’s *t*-test.

**FIGURE 5:**
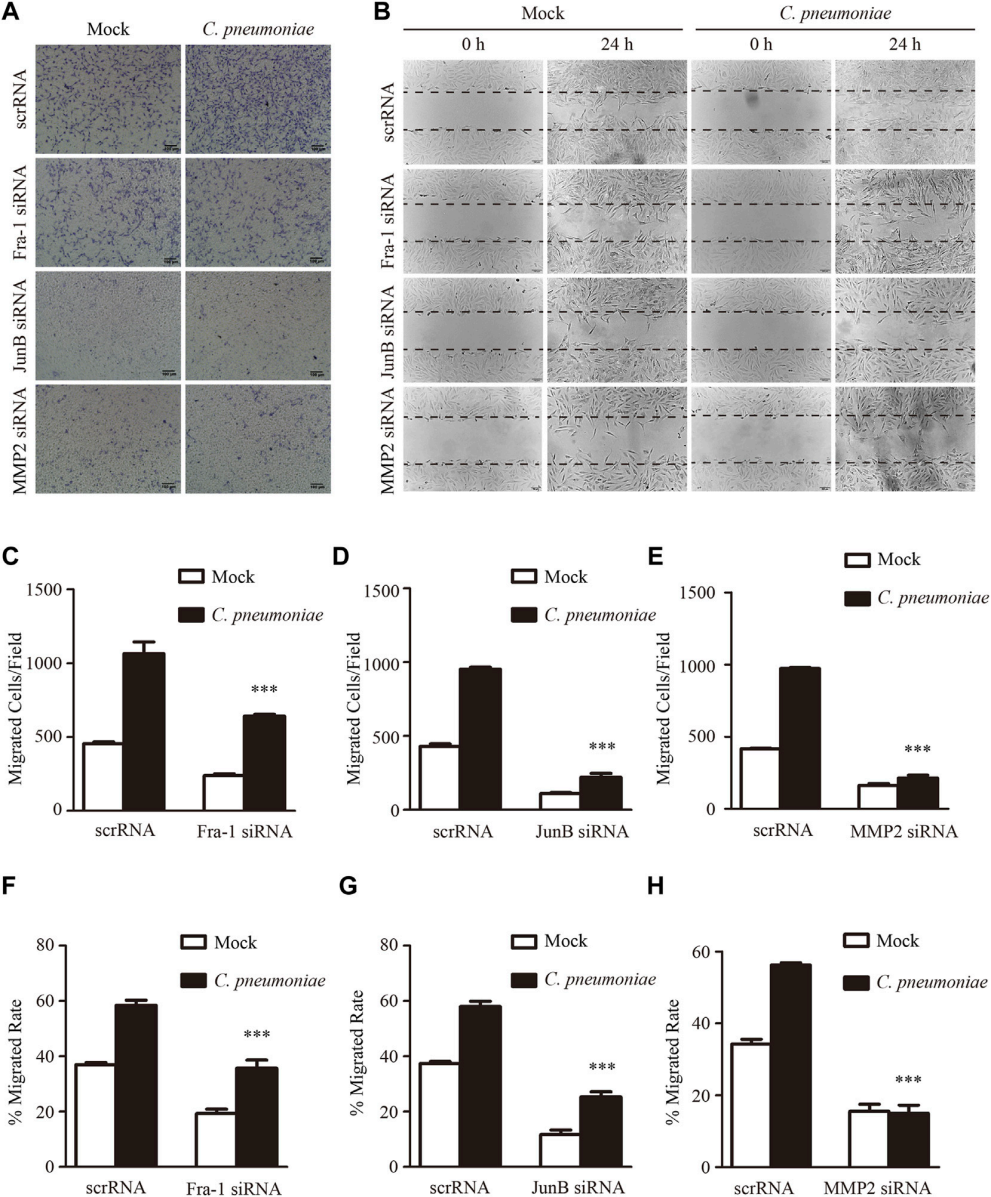
*C. pneumoniae* infection induces VSMC migration through Fra-1, JunB and MMP2. **(A,B)** Representative images of Transwell assays **(A)** and wound healing assays **(B)** for *C. pneumoniae* infection-induced migration of VSMCs pretreated with Fra-1 specific small interfering RNA (siRNA) or JunB specific siRNA or MMP2 specific siRNA or scrRNA. **(C–E)** The quantitative results for migrated VSMCs/field pretreated with the indicated specific siRNA. ***p < 0.001, compared with scramble siRNA (scrRNA) and *C. pneumoniae* infection group, as analyzed by Student’s *t*-test. **(F–H)** The quantitative results for the area of wound closure were detected at 24 h after scratch. VSMCs were pretreated with the indicated specific siRNA. ***p < 0.001, compared with scrRNA and *C. pneumoniae* infection group, as analyzed by Student’s *t*-test.

To identify the molecular mechanism of *C. pneumoniae* infection-induced VSMC migration, we checked the proteins involved in this process by using quantitative proteomic analysis. We found that the expressions of JunB, Fra-1 and MMP2 were significantly upregulated after *C. pneumoniae* infection (Figure 4A). And these results were then successfully verified by Western blot (Figures 4B–E). Furthermore, in ApoE^‐/‐^ mice model of *C. pneumoniae* infection-induced atherosclerosis, the expressions of JunB, Fra-1 and MMP2 in VSMCs in atherosclerotic lesions were also increased compared with mock infected ApoE^‐/‐^ mice (Figures 4F–I). To explore the roles of JunB, Fra-1 and MMP2 in *C. pneumoniae* infection-induced VSMC migration, we examined the effects of *C. pneumoniae* infection on the migratory capacity of VSMCs *in vitro* using wound healing and Transwell assays. The increased migratory capacity of VSMCs induced by *C. pneumoniae* infection was inhibited by either JunB or Fra-1 or MMP2 specific siRNA (Figures 5A–G).

### mtROS Increase MMP2 Expression Through Promoting the Formation of JunB-Fra-1 Complex

**FIGURE 6:**
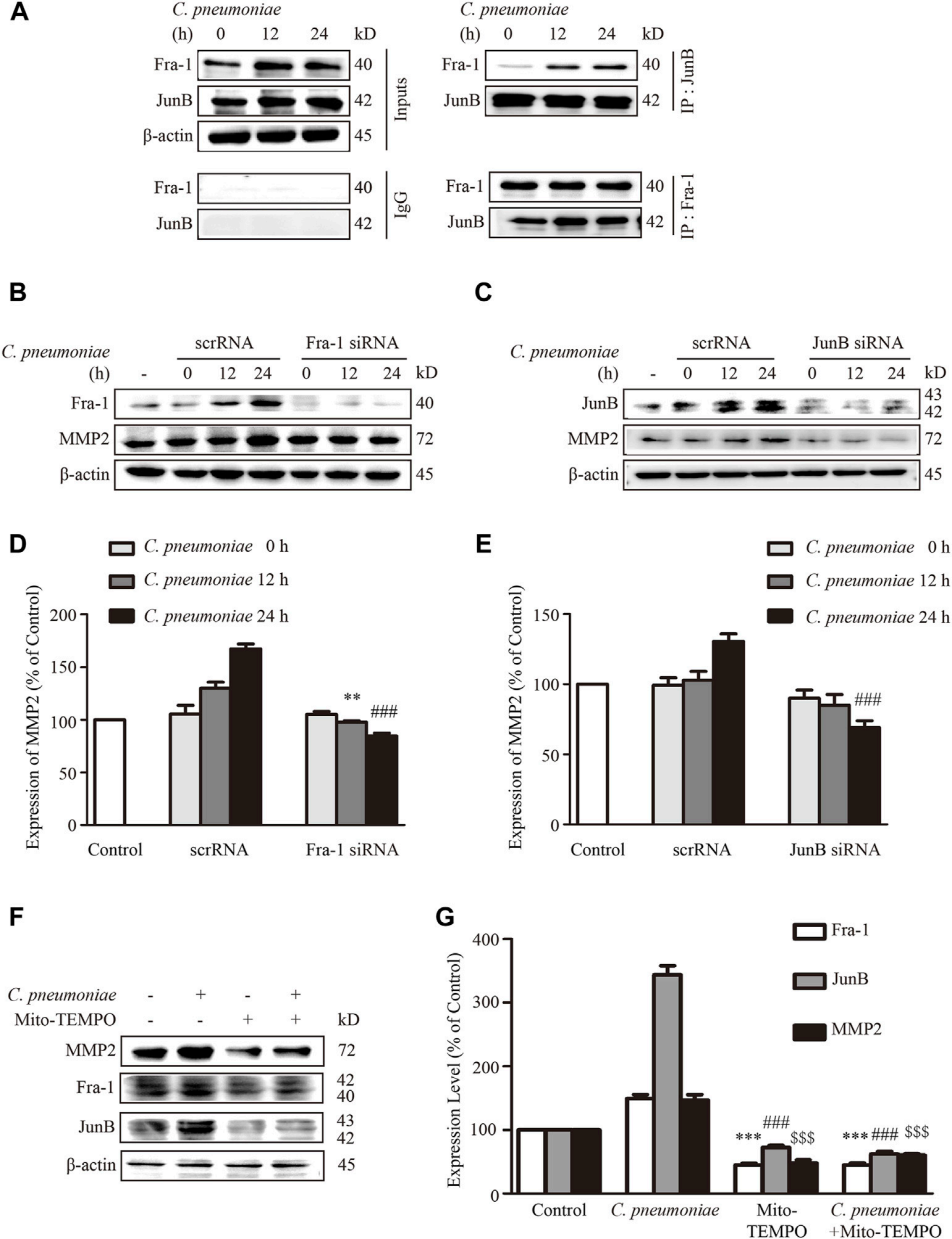
*C. pneumoniae* infection increases MMP2 expression through activating JunB and Fra-1 by mtROS. **(A)** VSMCs were infected with *C. pneumoniae* (5 × 10^5^ IFU) for 24 h. Cell lysates were immunoprecipitated with anti-Fra-1 antibody or anti-JunB antibody, and then Western blot was performed. IP, immunoprecipitation. Inputs were used as controls. **(B)** Representative Western blot images of Fra-1 and MMP2 from VSMCs pretreated with Fra-1 specific siRNA and infected with *C. pneumoniae* for the indicated timepoints. **(D)** The expression ratio of MMP2 to β-actin from three independent experiments is presented. **p < 0.01, compared with *C. pneumoniae* infection 12 h and scrRNA groups, as analyzed by Student’s *t*-test. ^###^ *p* < 0.001, compared with *C. pneumoniae* infection 24 h and scrRNA group, as analyzed by Student’s *t*-test. **(C)** Representative Western blot images of JunB and MMP2 from VSMCs pretreated with JunB specific siRNA and infected with *C. pneumoniae* for the indicated timepoints. **(E)** The expression ratio of MMP2 to β-actin from three independent experiments is presented. ^###^ *p* < 0.001, compared with *C. pneumoniae* infection 24 h and scrRNA group, as analyzed by Student’s *t*-test. **(F)** Representative Western blot images of Fra-1, JunB, and MMP2 from VSMCs pretreated with Mito-TEMPO and with or without *C. pneumoniae* infection. **(G)** The expression ratio of Fra-1, JunB and MMP2 to β-actin from three independent experiments is presented. ***p < 0.001, compared with *C. pneumoniae* infection 24 h and Fra-1 groups, as analyzed by Student’s *t*-test. ^###^ *p* < 0.001, compared with *C. pneumoniae* infection 24 h and JunB group, as analyzed by Student’s *t*-test. ^$$$^ *p* < 0.001, compared with *C. pneumoniae* infection 24 h and MMP2 group, as analyzed by Student’s *t*-test.

JunB and Fra-1 potentiate their transcriptional activity only when they form dimer (called AP-1). This promotes us to explore the potential interaction between JunB and Fra-1 in *C. pneumoniae*-infected VSMCs. The complex formation was identified by immunoprecipitation of both JunB followed by Fra-1 immunoblot and Fra-1 followed by JunB immunoblot (Figure 6A). In cardiac cells, a functional AP-1 site regulates MMP-2 transcription through interactions with JunB-Fra1 heterodimer (Bergman et al., 2003). Hence, we used JunB or Fra-1 specific siRNA to suppress the expression of JunB or Fra-1 to examine whether they affected the expression of MMP2 in the infected VSMCs. Western blot analysis showed that the knockdown of JunB or Fra-1 decreased MMP2 protein expression (Figures 6B–E). Increased expressions of JunB, Fra-1 and MMP2 induced by *C. pneumoniae* infection were all inhibited by Mito-TEMPO (Figures 6F,G).

### TLR2 Regulates mtROS-Related Signal Axis During *C. pneumoniae* Infection-Induced VSMC Migration and Atherosclerosis Development

**FIGURE 7:**
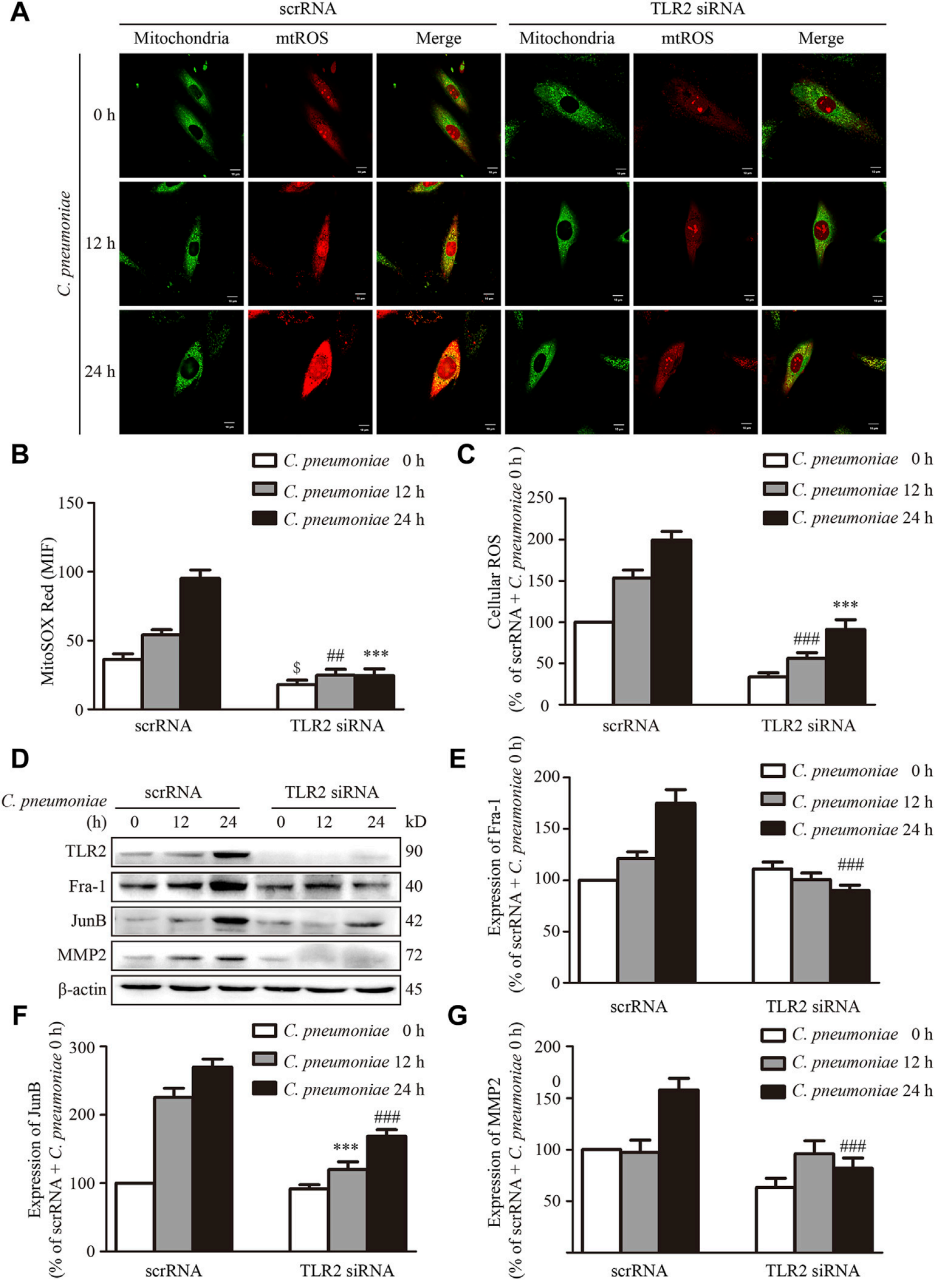
TLR2 regulates the expressions of Fra-1, JunB and MMP2 in VSMCs after *C. pneumoniae* infection *in vitro*. VSMCs were pretreated with TLR2 specific siRNA and infected with *C. pneumoniae* for the indicated timepoints. **(A)** Representative confocal microscopy images of VSMCs were stained with MitoSOX Red and Mito-Tracker Green after *C. pneumoniae* infection. Scale: 10 μM. **(B)** Quantification of MitoSOX Red fluorescence intensity in VSMCs. ^$^ *p* < 0.05, compared with scrRNA + *C. pneumoniae* infection 0 h group, as analyzed by Student’s *t*-test. ^##^ *p* < 0.01, compared with scrRNA + *C. pneumoniae* infection 12 h group, as analyzed by Student’s *t*-test. ***p < 0.001, compared with scrRNA + *C. pneumoniae* infection 24 h group, as analyzed by Student’s *t*-test. **(C)** Quantification of the level of cellular ROS in VSMCs infected with *C. pneumoniae* for the indicated timepoints. ^##^ *p* < 0.01, compared with scrRNA + *C. pneumoniae* infection 12 h group, as analyzed by Student’s *t*-test. ***p < 0.001, compared with scrRNA + *C. pneumoniae* infection 24 h group, as analyzed by Student’s *t*-test. **(D)** Representative Western blot images of TLR2, Fra-1, JunB and MMP2 from VSMCs pretreated with TLR2 specific siRNA and infected with *C. pneumoniae* for the indicated timepoints. **(E–G)** The expression ratio of Fra-1 **(E)** or JunB **(F)** or MMP2 **(G)** to β-actin from three independent experiments is presented. ***p < 0.01, compared with scrRNA + *C. pneumoniae* infection 12 h group, as analyzed by Student’s *t*-test. ^###^ *p* < 0.001, compared with scrRNA + *C. pneumoniae* infection 24 h group, as analyzed by Student’s *t*-test.

**FIGURE 8:**
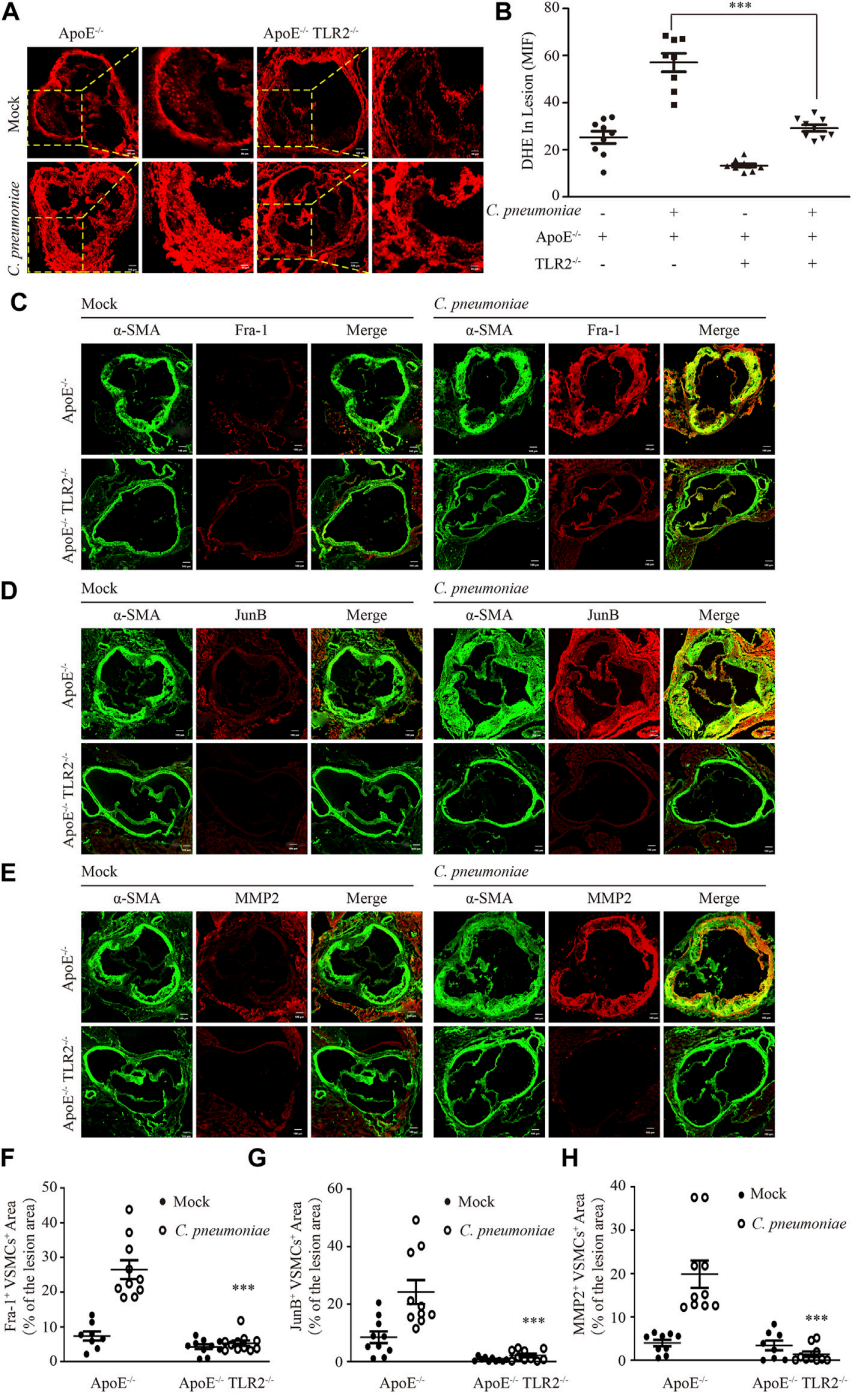
TLR2 regulates the expression of Fra-1, JunB and MMP2 in VSMCs after *C. pneumoniae* infection *in vivo*. **(A)** The ROS level was assessed by DHE in the lesions of aortic sinus from ApoE^‐/‐^ and ApoE^‐/‐^TLR2^‐/‐^ mice fed a Western diet for 6 weeks with or without *C. pneumoniae* infection. *n* = 8–11. Scale bar: 100 μM (Left), 50 μM (Right). **(B)** Quantification of DHE fluorescence intensity in the lesions of aortic sinus from ApoE^‐/‐^ and ApoE^‐/‐^TLR2^‐/‐^ mice fed a Western diet for 6 weeks with or without *C. pneumoniae* infection. ***p < 0.001, compared with *C. pneumoniae*-infected ApoE^‐/‐^ mice groups, as analyzed by Student’s *t*-test. **(C–E)** The expression of Fra-1 **(C)** or JunB **(D)** or MMP2 **(E)** in VSMCs in atherosclerotic lesions of ApoE^‐/‐^ and ApoE^‐/‐^TLR2^‐/‐^ mice fed a Western diet for 6 weeks with or without *C. pneumoniae* infection. Scale bar: 100 μM. **(F–H)** Quantification of active Fra-1^+^ **(F)**, JunB^+^ **(G)** and MMP2^+^ **(H)** VSMCs in the lesions of aortic sinus from ApoE^‐/‐^ and ApoE^‐/‐^TLR2^‐/‐^ mice fed a Western diet for 6 weeks with or without *C. pneumoniae* infection. ***p < 0.001, compared with *C. pneumoniae*-infected ApoE^‐/‐^mice groups, as analyzed by Student’s *t*-test.

Our previous studies showed that TLR2 was required for *C. pneumoniae* infection-induced VSMC migration (Beibei Wang et al., 2017; Miao et al., 2020). As a by-product of mitochondrial oxidative phosphorylation, mtROS is a predominant type of cellular ROS (Hengdao Liu et al., 2019) and plays a role in cell migration (Chen et al., 2020). We suppressed the expression of TLR2 by the specific siRNA *in vitro* to investigate whether TLR2 participates *C. pneumoniae* infection-induced VSMC migration by mtROS-related signal axis, and found that the levels of mtROS, cellular ROS and the expressions of JunB, Fra-1 and MMP2 were significantly reduced (Figures 7A–G), revealing the possible of TLR2/mtROS/JunB-Fra-1/MMP2 signal axis in *C. pneumoniae*-infected VSMCs. TLR2 has also been shown to have an important role in atherosclerosis (Naiki et al., 2008; Miao et al., 2020). Whether TLR2/mtROS/JunB-Fra-1/MMP2 signal axis participates in *C. pneumoniae* infection-induced atherosclerosis development remains unknown. Accordingly, we established *in vivo C. pneumoniae* infection-induced atherosclerosis models using ApoE^‐/‐^ and ApoE^‐/‐^TLR2^‐/‐^ mice, and found that the levels of ROS in atherosclerotic lesions were reduced by approximately 48.77% in ApoE^‐/‐^TLR2^‐/‐^ mice with *C. pneumoniae* infection compared with *C. pneumoniae-*infected ApoE^‐/‐^ mice (Figures 8A,B). And the decreases in the expressions of Fra-1, JunB and MMP2 in VSMCs were approximately 79.92%, 91.01% and 92.95% in the infected ApoE^‐/‐^TLR2^‐/‐^ mice compared with ApoE^‐/‐^ mice with *C. pneumoniae* infection (Figures 8C–H), suggesting that TLR2/mtROS/JunB-Fra-1/MMP2 signal axis plays a crucial role in *C. pneumoniae* infection-induced atherosclerosis development.

**SCHEME 1:**
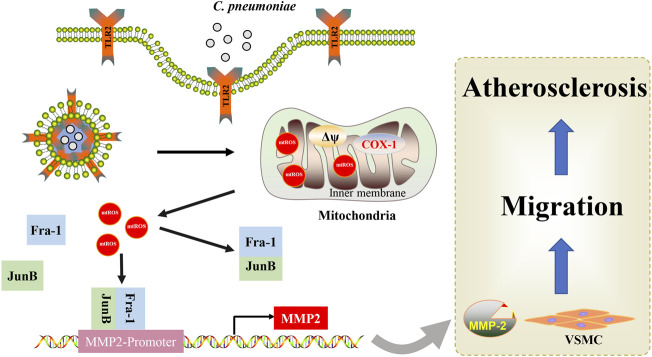
Mechanistic scheme of *C. pneumoniae* infection-promoted VSMC migration and atherosclerosis. A simple mechanism diagram illustrates that *C. pneumoniae* infection leads to mtROS accumulation through TLR2, and then activates JunB-Fra-1 to increase the expression of MMP2 to enhance VSMC migration and atherosclerotic lesion formation.

## Discussion

Accumulating studies have shown that mitochondrial dysfunctions are associated with atherosclerosis (Wei et al., 2018; Tyrrell et al., 2020). Recent evidences suggest that mitochondria-targeted antioxidant therapies to eliminate mtROS may have great promise in the prevention and treatment of atherosclerosis (Moss and Ramji, 2016; Atkinson et al., 2018). Our previous study showed that *C. pneumoniae* infection facilitated VSMC migration and promoted atherosclerotic lesion formation (Miao et al., 2020). However, whether mtROS participates in this process remains unknown. In the current study, the results showed the increase of ROS in VSMCs in atherosclerotic lesions after *C. pneumoniae* infection in ApoE^‐/‐^ mice. Our further studies found that eliminating mtROS by Mito-TEMPO could suppress *C. pneumoniae* infection-induced VSMC migration, proving that mtROS had significant effects on VSMC migration after *C. pneumoniae* infection. But how does mtROS participate in *C. pneumoniae* infection-induced VSMC migration and even atherosclerosis?

AP-1 is a menagerie of dimeric basic region-leucine zipper proteins composed of June, Fos, Maf and ATF sub-families (Bai et al., 2018). The diverse cellular responses were mediated by the specific subunit composition of the AP-1 complex (Eferl and Wagner, 2003). Our previous results demonstrated that c-Fos may have an influence on VSMC migration after *C. pneumoniae* infection (Zheng et al., 2019). And other studies showed that mtROS was a signaling mediator to drive the activation of AP-1 (Li et al., 2016; Yu et al., 2019) and promoted VSMC migration (Park et al., 2015; Chou et al., 2019). In T helper [T(H)17] cell, B-ATF (belong to ATF family) had a critical role in T(H)17 differentiation (Schraml et al., 2009). However, in our model of VSMC infected with *C. pneumoniae*, Fra-1 and JunB formed a heterodimer to enhance their transcriptional activity, but not other sub-families. When we reduced the level of mtROS, the expressions of Fra-1 and JunB were both decreased, suggesting that *C. pneumoniae* infection activates Fra-1 and JunB by mtROS to promote VSMC migration. It is interesting to note that the expression and activity of AP-1 family proteins c-Jun, c-Fos and ATF-2 were all upregulated in a time- and dose-dependent manner when Hela cells were infected with *C. pneumoniae* (Bakiri et al., 2002). But, in human coronary artery endothelial cells, Fos, FosB and JunB dominated the transcription factor network initiated by *C. pneumoniae* infection (Wang et al., 2013). These results indicate that the composition of the AP-1 complex is different in different cell types in spite of the same stimulus.

In addition, we also found that silencing JunB or Fra-1 significantly decreased the migration of VSMCs after *C. pneumoniae* infection. But, the mechanism by which JunB and Fra-1 affect VSMC migration still needs to be elucidated. The extracellular matrix (ECM) is a highly dynamic structure that is present in all tissues. The migration of cells regulated by ECM involves quantitative and qualitative changes, which are mediated by specific enzymes (Bonnans et al., 2014; Yamada and Sixt, 2019). MMPs are responsible for ECM degradation and remodeling (Hynes, 2009). And a number of members of MMPs are considered to have a close relationship with VSMC migration, such as MMP2, MMP3, MMP9 (Ma et al., 2015; Meng et al., 2020). Our previous data suggested that *C. pneumoniae* infection induced VSMC migration through MMP3 and MMP9 *via* PI3K (Ma et al., 2015). In the present study, we found that JunB-Fra-1 led to enhanced activity of MMP2 in VSMCs infected with *C. pneumoniae*. It was reported that a functional AP-1 site regulated MMP-2 transcription through interactions with JunB-Fra1 heterodimers in cardiac cells (Bergman et al., 2003). It is at least partly suggested that the activation of JunB and Fra-1 inducing MMP2 upregulation has a role in VSMC migration after *C. pneumoniae* infection. Based on these data, it is reasonable to conclude that eliminated mtROS significantly suppresses *C. pneumoniae* infection-induced increases in the expressions of JunB, Fra-1 and MMP2 as well as VSMC migration. Accordingly, reducing the level of mtROS to block *C. pneumoniae* infection-induced VSMC migration-related signal axis, JunB-Fra-1/MMP2 signal axis, may become a potential treatment target for *C. pneumoniae* infection-induced atherosclerosis. A study from Chen et al. who found that astaxanthin decreased the level of cellular ROS to attenuate hypertensive vascular remodeling by mitigating VSMC migration also supported our results, to some extent (Chen et al., 2020).

TLR2 has been demonstrated to regulate VSMC migration through P38/MAKP, PI3K/AKT (Wu et al., 2016) and CXCR4/FAK signal pathway (Miao et al., 2020). Studies have also reported that TLR2 could affect mtROS production (West et al., 2011; Li et al., 2020a), changing mitochondrial energy metabolism (Cao et al., 2019) and regulating expression of manganese-dependent mitochondrial enzymes (You Dong Liu et al., 2019). An unavoidable consequence of mitochondrial dysfunction is the accumulation of mtROS, which was considered as important second-messenger signals within cells (Kim et al., 2019). In breast cancer cells (Cheriyath et al., 2018), microvascular endothelial cells (Suresh et al., 2019) and microglia (Miyake et al., 2015), cell migration closely correlated with mtROS. But whether and how can activated TLR2 induce mtROS accumulation and activate mtROS-mediated VSMC migration-related signal axis to promote VSMC migration and atherosclerotic lesion formation after *C. pneumoniae* infection? Based on these questions, we used ApoE^‐/‐^TLR2^‐/‐^ mice and primary VSMCs to demonstrate that TLR2 participated in cell migration and atherosclerotic lesion formation through regulating the level of mtROS after *C. pneumoniae* infection. Therefore, our results further improve the understanding of the mechanism of TLR2 in VSMC migration.

In summary, this study demonstrated that TLR2 was required for the accumulation of mtROS after *C. pneumoniae* infection and highlighted the activation of JunB-Fra-1 by mtROS to promote the expression of MMP2 in the infection-induced VSMC migration and atherosclerotic lesion formation. We characterized the anti-oxidation strategy in blocking mtROS-mediated VSMC migration after *C. pneumoniae* infection, which may help to guide future therapeutic direction to manage *C. pneumoniae* infection-induced atherosclerosis.

## Data Availability

The original contributions presented in the study are included in the article/Supplementary Material, further inquiries can be directed to the corresponding author. The mass spectrometry proteomics data have been deposited to the ProteomeXchange Consortium, internal ID is #565452.
