# Supplementary material for: Chlamydia pneumoniae Infection Induces Vascular Smooth Muscle Cell Migration and Atherosclerosis Through Mitochondrial Reactive Oxygen Species-Mediated JunB-Fra-1 Activation
Source: Front Cell Dev Biol. 2022 Apr 12;10:879023. doi: 10.3389/fcell.2022.879023 (PMC9039263; doi:10.3389/fcell.2022.879023)
Supplement: Supplementary file 1 [file DataSheet1.docx]

Supplementary Material

## Supplementary Figure


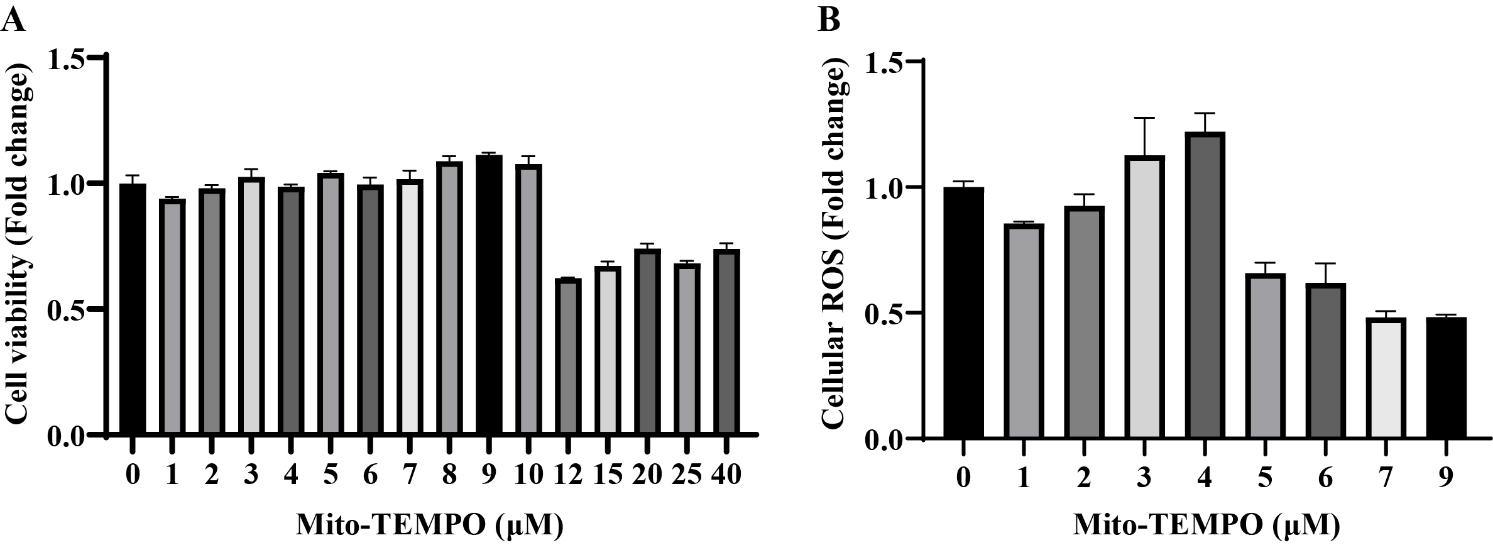


**Supplementary Figure.** Dose-dependent effects of Mito-TEMPO on VSMC viability and cellular ROS. **A.** VSMC viability was assessed by CCK-8 kit after cells were treated with different dose of Mito-TEMPO for 24 h. **B.** Elimination efficiency of different dose of Mito-TEMPO on the cellular ROS in VSMCs was measured by using CM-H2DCFDA (10 μM).

# Supplementary Table

Table 1 Summary of the changed proteins in mock infection vs. *C. pneumoniae* infection in VSMC.

| Accession | Gene name | Unique Peptides | MW [kDa] | Ratio |
| --- | --- | --- | --- | --- |
| Q6XS76 | Nos2 | 1 | 130.6 | 1.768106 |
| P0CG51 | Ubb | 1 | 34.3 | 1.224884 |
| Q91W30 | Akr1b8 | 18 | 36.2 | 1.310275 |
| D3ZF39 | Uap1 | 27 | 58.4 | 1.203569 |
| Q9ERB4 | Vcan | 13 | 299.8 | 1.73129 |
| P11883 | Aldh3a1 | 19 | 50.3 | 1.258343 |
| F1MA18 | Ybx3 | 1 | 38.8 | 1.225277 |
| D4A8Y6 | Vcan | 2 | 332.2 | 1.572087 |
| Q5U2U5 | Plin2 | 16 | 46.2 | 1.611086 |
| P04906 | Gstp1 | 7 | 23.4 | 1.244294 |
| Q71SA3 | Thbs1 | 32 | 129.6 | 1.626798 |
| Q498M9 | LOC303448 | 1 | 36.1 | 2.294786 |
| P06762 | Hmox1 | 9 | 33.0 | 1.402131 |
| Q4KMC4 | Gfpt2 | 18 | 77.0 | 1.514866 |
| P08011 | Mgst1 | 3 | 17.5 | 1.207597 |
| A0A0G2K1S6 | Me1 | 1 | 63.8 | 1.275867 |
| Q6DQ97 | / | 6 | 16.3 | 1.928203 |
| G3V7Z4 | Serpine2 | 8 | 44.0 | 1.509827 |
| A0A0G2KAP1 | Ero1b | 1 | 53.4 | 1.264761 |
| Q6QI48 | Scp2 | 1 | 72.5 | 1.201033 |
| G3V843 | F2 | 6 | 70.3 | 1.796084 |
| D3ZGB1 | Nfat5 | 6 | 167.1 | 1.201861 |
| P08635 | Olah | 9 | 29.5 | 1.209105 |
| A0A0G2JSH5 | Alb | 4 | 68.7 | 2.422169 |
| F1LMZ4 | Gfm2 | 3 | 85.9 | 1.407881 |
| E9PU64 | Scin | 14 | 80.1 | 1.306044 |
| D3ZT94 | Ptx3 | 8 | 42.0 | 1.430781 |
| D3ZFH5 | Itih2 | 5 | 92.3 | 1.588987 |
| A0A0G2JSY8 | Junb | 3 | 35.8 | 1.401793 |
| F7FPF5 | Sp100 | 5 | 38.0 | 1.475805 |
| Q6AYA6 | / | 4 | 20.9 | 1.228886 |
| V9GZL5 | / | 1 | 8.6 | 1.314135 |
| Q62738 | Fosl2 | 2 | 26.1 | 1.200241 |
| M0R5T8 | RGD1566189 | 3 | 20.7 | 1.249164 |
| D3ZC87 | Maff | 1 | 17.0 | 1.282984 |
| P62329 | Tmsb4x | 4 | 5.0 | 1.201719 |
| A0A0G2K2S2 | Slc2a1 | 4 | 53.8 | 1.246754 |
| Q5I0P8 | Ptges | 5 | 17.1 | 1.464012 |
| Q76MX4 | Mafg | 1 | 20.5 | 1.258904 |
| Q63041 | A1m | 4 | 167.0 | 2.227304 |
| D3ZY40 | Pcf11 | 3 | 172.6 | 1.335275 |
| P63312 | Tmsb10 | 1 | 5.0 | 1.339203 |
| Q64599 | LOC286987 | 5 | 24.1 | 1.888563 |
| D3ZQ25 | Fbln1 | 4 | 78.0 | 1.752388 |
| Q5XI63 | Kifc1 | 5 | 76.1 | 1.242319 |
| A0A0A0MXZ0 | Isca1 | 2 | 14.1 | 1.342209 |
| A0A0G2K3W2 | F5 | 2 | 190.9 | 1.529832 |
| B2RYV8 | Mrpl52 | 1 | 13.7 | 1.44916 |
| A2VD07 | Cyp4f5 | 1 | 60.7 | 1.211483 |
| A0A0G2JV52 | C4a | 3 | 150.8 | 2.509919 |
| Q68G30 | Klc3 | 1 | 55.6 | 1.378008 |
| Q6TXF2 | Anxa3 | 1 | 28.1 | 1.286283 |
| D3Z962 | Tacc3 | 2 | 65.1 | 1.204665 |
| P70566 | Tmod2 | 1 | 39.5 | 2.204209 |
| D4AEB0 | Hdac3 | 3 | 38.0 | 1.200821 |
| F1M400 | Ankrd28 | 2 | 115.9 | 1.249783 |
| D4A753 | Ell | 3 | 67.3 | 1.201461 |
| Q5RKG5 | RGD1309594 | 1 | 28.0 | 1.412646 |
| G3V719 | Plscr4 | 2 | 36.3 | 1.216664 |
| P30152 | Lcn2 | 3 | 22.5 | 1.405571 |
| A0A0G2K9H5 | Nod1 | 1 | 104.1 | 1.425003 |
| A0A1S7IVG9 | Vkorc1 | 1 | 17.7 | 1.239331 |
| D3Z8B0 | Cox16 | 2 | 12.2 | 1.259937 |
| Q5BJP5 | Tmem230 | 2 | 13.2 | 1.302728 |
| P10158 | Fosl1 | 3 | 30.1 | 1.757154 |
| Q6XVN8 | Map1lc3a | 1 | 14.3 | 1.281784 |
| A0A0G2JW37 | RGD1561318 | 1 | 34.8 | 1.268703 |
| P97682 | Esm1 | 2 | 20.1 | 1.218689 |
| A0A0G2K0E2 | RGD1311745 | 1 | 15.9 | 1.779197 |
| P06238 | A2m | 3 | 163.7 | 3.250567 |
| B2GV52 | Bloc1s5 | 2 | 21.5 | 1.484839 |
| M0RBJ7 | C3 | 5 | 186.1 | 1.345527 |
| Q641X5 | Gpat4 | 2 | 43.2 | 1.262654 |
| A0A0G2JSV6 | Hba-a2 | 3 | 15.3 | 2.18201 |
| D3ZJB2 | Ginm1 | 2 | 36.3 | 1.31491 |
| P02767 | Ttr | 2 | 15.7 | 1.355494 |
| G3V9I9 | Srek1 | 2 | 69.2 | 1.386397 |
| D3ZAB6 | Rnf219 | 2 | 80.0 | 1.247001 |
| B1WBV2 | Sfrp2 | 2 | 33.5 | 1.214773 |
| Q5M807 | Rnf5 | 1 | 19.8 | 1.332217 |
| Q7TNK0 | Serinc1 | 2 | 50.5 | 1.218678 |
| D4AE80 | Dcp1a | 3 | 65.1 | 1.200624 |
| D4ABG0 | Fam83d | 2 | 64.3 | 1.388795 |
| A1A5P7 | Plekha1 | 1 | 38.0 | 1.256414 |
| Q5BK07 | Ccdc43 | 2 | 25.0 | 1.309409 |
| F1MAB8 | Kif11 | 6 | 118.2 | 1.289502 |
| F6PUS4 | Sh3d21 | 1 | 59.5 | 1.405218 |
| M0R8C6 | Slc14a1 | 5 | 42.2 | 1.21637 |
| Q5RJQ7 | Sdhaf2 | 1 | 19.5 | 1.349159 |
| Q4KMA3 | LOC361985 | 2 | 28.7 | 1.317766 |
| A0A0G2JX37 | Slc2a3 | 2 | 52.5 | 1.221087 |
| B1WC13 | LOC100151767 | 2 | 33.7 | 1.290057 |
| D3ZK16 | Ice1 | 1 | 240.9 | 1.200124 |
| F1M3X5 | Mroh6 | 1 | 78.0 | 2.176729 |
| A0A0G2K7Y7 | Kif23 | 5 | 101.9 | 1.211089 |
| P24090 | Ahsg | 2 | 38.0 | 1.805243 |
| O70248 | Apba3 | 3 | 60.8 | 1.22145 |
| E9PU78 | Bcl2l1-ps1 | 1 | 19.0 | 1.218749 |
| Q6R5Q0 | Tfap2c | 1 | 49.0 | 2.590638 |
| D3ZBS2 | Itih3 | 2 | 99.0 | 2.02957 |
| Q5XIB6 | Apol9a | 3 | 33.7 | 1.308909 |
| M0RBE0 | LOC102552166 | 1 | 122.0 | 1.220073 |
| B0K036 | Sdhaf1 | 1 | 13.3 | 1.33934 |
| D3ZHV3 | Mt1m | 2 | 6.1 | 1.309021 |
| R9PXX6 | Pan2 | 1 | 134.4 | 2.298732 |
| M0R8I8 | Birc5 | 1 | 16.5 | 1.218927 |
| P33436 | Mmp2 | 1 | 74.1 | 1.376923 |
| A0A0G2JWD4 | Lrrc74a | 1 | 51.7 | 1.209418 |
| Q9QZX9 | Cep19 | 1 | 19.1 | 1.510655 |
| D3ZMK9 | Prag1 | 1 | 147.6 | 1.391872 |
| Q6PCT7 | Tdrkh | 1 | 32.4 | 1.256715 |
| A0A0G2JSM1 | Selenos | 2 | 21.1 | 1.267084 |
| D3ZAB1 | Ltf | 1 | 79.8 | 1.515883 |
| P59647 | Fxyd5 | 1 | 19.1 | 1.323994 |
| Q5M7W7 | Pars2 | 3 | 53.3 | 1.221648 |
| D4AAE6 | Rab20 | 1 | 25.8 | 1.364243 |
| F1LWB9 | Ankrd50 | 1 | 155.5 | 1.392242 |
| Q498S0 | Chst12 | 2 | 49.2 | 1.238387 |
| Q5PPN2 | Rpp25 | 1 | 20.9 | 1.26934 |
| D4ACF5 | Brd9 | 1 | 67.0 | 1.700818 |
| Q5PT50 | Slc10a7 | 1 | 37.3 | 1.263081 |
| Q9QYW4 | Gdf9 | 1 | 49.6 | 1.854724 |
| G3V8I1 | Alpi | 1 | 58.3 | 1.357856 |
| Q5M7T5 | Serpinc1 | 4 | 52.2 | 2.113063 |
| Q5BK21 | Tm7sf2 | 2 | 46.4 | 1.207742 |
| D3ZBC9 | Maats1 | 1 | 90.0 | 1.863536 |
| P02650 | Apoe | 1 | 35.7 | 1.652458 |
| Q06BI3 | Caln1 | 1 | 24.8 | 1.963315 |
| Q5FVQ0 | Slc39a8 | 1 | 50.1 | 2.348336 |
| P09034 | Ass1 | 1 | 46.5 | 1.235302 |
| Q4KLH6 | Cep162 | 1 | 160.9 | 4.40722 |
| O09178 | Ampd3 | 1 | 88.4 | 1.217899 |
| Q68FT3 | Pyroxd2 | 1 | 62.8 | 1.234719 |
| Q66HG0 | Rnf13 | 2 | 42.6 | 1.306663 |
| F1M5M3 | Tex14 | 1 | 162.3 | 1.494019 |
| P02680 | Fgg | 2 | 50.6 | 1.240378 |
| P32182 | Foxa2 | 1 | 48.5 | 1.220205 |
| Q6P730 | Dab2ip | 1 | 109.9 | 1.346699 |
| D3ZY60 | Plekha8 | 1 | 58.1 | 1.806603 |
| Q5HZF2 | Wbp4 | 1 | 42.1 | 1.201859 |
| P59241 | Aurka | 1 | 44.8 | 1.2204 |
| Q9JKF8 | Rasd1 | 1 | 31.7 | 1.347341 |
| Q9R010 | Cib1 | 1 | 21.8 | 1.250729 |
| Q5U3Y0 | Klhdc10 | 1 | 45.4 | 1.300118 |
| O09027 | Ackr2 | 1 | 43.3 | 1.793452 |
| Q6P6R7 | Sgsm3 | 1 | 85.1 | 1.290057 |
| P0C5J5 | Wfikkn1 | 1 | 59.9 | 1.232893 |
| Q63046 | Runx1 | 1 | 48.5 | 1.252215 |
| Q5XIV0 | Fam46c | 1 | 44.8 | 1.302673 |
| P23640 | Rab27a | 1 | 25.1 | 1.269851 |
| P01161 | Nppa | 1 | 16.5 | 1.201363 |
| A0A0H2UI11 | Rpl39 | 1 | 6.3 | 1.444627 |
| B5MEV3 | Slc52a2 | 1 | 46.9 | 1.665363 |
| M0R410 | Rp1l1 | 2 | 200.3 | 2.270596 |
| D3ZKD9 | Mapt | 1 | 39.1 | 1.238115 |
| D3ZBB3 | Tmem47 | 1 | 20.0 | 1.25919 |
| Q4KM75 | Cd5l | 1 | 37.8 | 2.485673 |
| F1LQT1 | Adcy7 | 1 | 122.9 | 1.293334 |
| D4A3Z3 | Tbc1d24 | 1 | 62.8 | 1.323086 |
| M0R4S2 | Apod | 1 | 21.6 | 1.607164 |
| D3ZES2 | Trappc6b | 1 | 18.0 | 1.255829 |
| D4ACD3 | Usp25 | 2 | 121.5 | 1.336325 |
| D3ZT76 | Zfp13 | 1 | 44.5 | 1.342027 |
| M0R9R0 | Gprasp1 | 1 | 151.4 | 1.754453 |
| Q9Z217 | Gdf11 | 1 | 44.9 | 1.227188 |
| A0A0G2JSW3 | Hbb | 2 | 16.0 | 1.26126 |
| D3ZA68 | Twnk | 1 | 76.8 | 1.205918 |
| Q496Z8 | Lrp10 | 1 | 76.2 | 1.412553 |
| D4A060 | Snx30 | 1 | 49.4 | 1.437605 |
| G3V675 | Whrn | 1 | 98.2 | 1.471454 |
| A0A0H2UHA9 | Mmd | 1 | 26.2 | 1.2583 |
| A0A0G2JSP8 | Ckm | 1 | 43.0 | 1.924848 |
| D3ZF99 | Mrpl55 | 1 | 15.1 | 1.374146 |
| D3ZVH5 | Mdm2 | 1 | 51.0 | 1.213729 |
| G3V756 | Slc9a8 | 1 | 64.6 | 1.209624 |
| D4A9X9 | Cenpk | 1 | 35.0 | 1.316589 |
| B5DFG8 | Pygo2 | 1 | 41.1 | 1.451841 |
| B0BN24 | LOC100909485 | 1 | 29.5 | 1.287405 |
| B2RYU0 | Ndufb2 | 1 | 11.8 | 1.304236 |
| D3ZB49 | Osgin2 | 1 | 61.3 | 1.252002 |
| F1LPJ2 | Ryr3 | 1 | 549.9 | 1.474216 |
| D3ZDJ5 | Rrp36 | 1 | 28.4 | 1.872951 |
| D4ADM6 | Alpp | 1 | 57.2 | 1.732765 |
| D4ABM1 | Cyp2c7 | 1 | 56.2 | 2.025986 |
| F7FLS6 | Lzts2 | 1 | 72.5 | 1.341988 |
| C9DRP4 | pfkfb1 | 1 | 47.4 | 1.473063 |
| F1LZB9 | LOC689396 | 1 | 71.8 | 1.748606 |
| D3ZB56 | Ccdc66 | 1 | 91.8 | 1.627089 |
| M0R3W7 | Enpp7 | 1 | 49.9 | 1.783458 |
| O09182 | / | 1 | 69.0 | 1.225888 |
| D4A709 | Tubgcp6 | 1 | 197.0 | 1.520437 |
| A0A0G2JWA2 | Plcg2 | 1 | 147.5 | 1.287355 |
| B2RZB4 | Metap1d | 1 | 36.8 | 1.471291 |
| G3V676 | Abcc5 | 2 | 160.2 | 1.219933 |
| Q5UDQ9 | Ppfibp2 | 1 | 61.8 | 1.27501 |
| F1M0M0 | Casz1 | 1 | 184.0 | 1.866179 |
| M0RDI0 | Cyp3a9 | 1 | 57.8 | 1.308912 |
| M0R890 | Abca6 | 1 | 183.9 | 1.206115 |
| Q4G066 | Gnptg | 1 | 34.0 | 1.272631 |
| D3ZIP1 | Kcnrg | 1 | 30.0 | 1.273654 |
| A0A0G2K715 | Cep290 | 1 | 289.5 | 1.216022 |
| D3ZK45 | Epb41l5 | 1 | 57.6 | 1.227517 |
| A0A0G2K230 | Dsc3 | 1 | 93.6 | 1.393821 |
| F6Q5G6 | Aplf | 1 | 56.2 | 1.267051 |
| F1LVL5 | Shroom4 | 1 | 164.5 | 1.438935 |
| D4A562 | Ift81 | 1 | 79.0 | 1.603325 |
| D3ZF29 | Dnase1l2 | 1 | 31.0 | 1.293862 |
| A0A0G2JZ58 | Sdccag8 | 1 | 77.2 | 1.374659 |
| F1M6U0 | Wwc1 | 1 | 124.4 | 1.960543 |
| F1M5M9 | Srgap3 | 1 | 124.4 | 1.436246 |
| A0A0G2K0F5 | Myh1 | 1 | 223.0 | 1.581924 |
| F1LR53 | Slc25a30 | 2 | 32.3 | 1.261289 |
| D3ZTH0 | Tmc5 | 1 | 87.8 | 3.013027 |
| A0A0G2JYJ5 | / | 2 | 60.4 | 1.231953 |
| M0R7M4 | Rad51ap2 | 1 | 111.0 | 1.327474 |
| A0A0G2JUP3 | Obscn | 3 | 814.9 | 1.9703 |
| A0A0G2JZX5 | Iqsec2 | 1 | 105.1 | 1.241099 |
| F2Z3S0 | Mis12 | 1 | 24.2 | 1.228215 |
| B2RYC5 | RGD1310209 | 1 | 81.6 | 1.661809 |
| A0A0G2K7Q4 | Hand2 | 1 | 23.3 | 1.569343 |
| A0A0G2K1U3 | RGD1307947 | 1 | 23.6 | 1.635869 |
| A0A0G2K972 | Tdrd15 | 1 | 228.0 | 2.953383 |
| A0A0H2UI24 | Rnf10 | 1 | 88.5 | 1.356656 |
| A0A1B0GWP7 | Lama3 | 1 | 98.4 | 1.80276 |
| A0A1B0GWT3 | Traf1 | 1 | 19.8 | 1.606031 |
| P70475 | Myt1l | 1 | 132.8 | 1.230442 |
| A0A1W2Q681 | Sfswap | 2 | 101.4 | 1.232471 |
| P15429 | Eno3 | 17 | 47.0 | 0.828824 |
| Q6IRK8 | Sptan1 | 1 | 282.2 | 0.810197 |
| P02454 | Col1a1 | 63 | 137.9 | 0.757103 |
| P02262 | / | 2 | 14.1 | 0.774121 |
| D4ACV3 | Hist2h2ac | 1 | 13.6 | 0.809529 |
| Q64598 | / | 2 | 14.2 | 0.809866 |
| G3V9G4 | Acly | 1 | 119.6 | 0.820709 |
| F1LND7 | Fdps | 14 | 40.8 | 0.820872 |
| G3V824 | Igf2r | 1 | 273.4 | 0.830332 |
| P53534 | Pygb | 1 | 96.1 | 0.820054 |
| P13668 | Stmn1 | 19 | 17.3 | 0.829693 |
| A0A0G2K931 | Psat1 | 2 | 40.5 | 0.721409 |
| P17425 | Hmgcs1 | 17 | 57.4 | 0.652856 |
| Q62967 | Mvd | 10 | 43.9 | 0.77311 |
| P36201 | Crip2 | 8 | 22.7 | 0.820178 |
| Q63312 | Phldb1 | 1 | 93.5 | 0.801565 |
| Q64654 | Cyp51a1 | 13 | 56.7 | 0.54334 |
| Q920L2 | Sdha | 20 | 71.6 | 0.828038 |
| Q66HF1 | Ndufs1 | 20 | 79.4 | 0.792802 |
| O35353 | Gnb4 | 1 | 37.3 | 0.816009 |
| P13852 | Prnp | 5 | 27.8 | 0.79602 |
| P63012 | Rab3a | 1 | 25.0 | 0.794532 |
| F1M8P6 | / | 2 | 27.7 | 0.620171 |
| A0A0G2K5E8 | NEWGENE_621351 | 9 | 120.0 | 0.752418 |
| O08628 | Pcolce | 13 | 50.2 | 0.739453 |
| D3ZS58 | Ndufa2 | 5 | 10.8 | 0.749209 |
| D3ZG43 | Ndufs3 | 5 | 30.2 | 0.812683 |
| Q5XIG5 | Gkap1 | 13 | 41.9 | 0.829536 |
| O35760 | Idi1 | 8 | 26.4 | 0.732804 |
| F1LRM7 | Col2a1 | 1 | 134.5 | 0.664306 |
| B5DF62 | Pak4 | 1 | 64.7 | 0.747895 |
| A0A0G2JYD8 | Capn5 | 7 | 73.2 | 0.82025 |
| Q641Y2 | Ndufs2 | 8 | 52.5 | 0.811173 |
| Q66H98 | Cavin2 | 10 | 46.4 | 0.802734 |
| A0A0G2JUK8 | Samd4a | 1 | 79.5 | 0.740361 |
| A0A0G2JX47 | Col5a1 | 1 | 178.0 | 0.829327 |
| Q63783 | NFI-A | 1 | 53.6 | 0.720528 |
| G3V9L9 | Clca4l | 3 | 100.0 | 0.828452 |
| Q5XIH3 | Ndufv1 | 4 | 50.7 | 0.763127 |
| G3V644 | Ndufv3 | 6 | 49.3 | 0.743248 |
| O35358 | / | 1 | 3.0 | 0.682325 |
| Q9JMI1 | Aacs | 11 | 75.0 | 0.792736 |
| D3ZCZ9 | LOC100912599 | 3 | 13.0 | 0.643587 |
| A9UMV9 | Ndufa7 | 5 | 12.5 | 0.656894 |
| Q63362 | Ndufa5 | 5 | 13.4 | 0.812406 |
| B2RZD6 | Ndufa4 | 5 | 9.3 | 0.75066 |
| F1LRJ6 | Pola1 | 1 | 152.4 | 0.819852 |
| A0A1W2Q6F8 | Ndufa10l1 | 5 | 40.5 | 0.757458 |
| P09606 | Glul | 8 | 42.2 | 0.819542 |
| Q5XIF3 | Ndufs4 | 3 | 19.7 | 0.629832 |
| D3ZE15 | LOC100911483 | 3 | 16.8 | 0.782283 |
| D3ZIX4 | H1fx | 4 | 20.5 | 0.808313 |
| Q5RJN0 | Ndufs7 | 3 | 23.9 | 0.701349 |
| P21913 | Sdhb | 11 | 31.8 | 0.738679 |
| D3ZXS5 | Dctd | 1 | 19.3 | 0.832287 |
| D4A4L1 | Sohlh1 | 2 | 38.0 | 0.772782 |
| A0A0G2JSX5 | Ednra | 2 | 48.2 | 0.773661 |
| Q6IFV4 | Krt13 | 1 | 47.7 | 0.774948 |
| D4A3V2 | Ndufa6 | 3 | 15.2 | 0.739403 |
| Q9QZD1 | Cxcl12 | 2 | 10.0 | 0.79587 |
| D4A987 | Arhgap31 | 1 | 155.4 | 0.719876 |
| P07340 | Atp1b1 | 3 | 35.2 | 0.737151 |
| D3ZMQ9 | Hltf | 1 | 110.0 | 0.822462 |
| M0RBH9 | Gas1 | 3 | 40.2 | 0.663294 |
| D3ZH39 | Ephb3 | 1 | 115.5 | 0.831196 |
| O35532 | Msmo1 | 3 | 34.9 | 0.66966 |
| Q62707 | / | 1 | 31.1 | 0.780009 |
| A0A0G2JW34 | Cisd3 | 5 | 14.4 | 0.765162 |
| P13941 | Col3a1 | 4 | 138.9 | 0.630059 |
| Q792Q4 | Cript | 4 | 11.3 | 0.832673 |
| Q63117 | Clk3 | 2 | 58.4 | 0.830867 |
| A0A0G2JV84 | Gpt2 | 3 | 60.2 | 0.832276 |
| M0RDU8 | Scd2 | 7 | 41.0 | 0.637536 |
| D3ZFW8 | Ccr1l1 | 1 | 40.7 | 0.762819 |
| Q641Z9 | Sdhc | 1 | 18.2 | 0.775437 |
| A0A0G2K3D7 | Zbtb1 | 1 | 73.0 | 0.747376 |
| D3Z9A5 | Btbd18 | 1 | 79.2 | 0.801515 |
| G3V960 | Gamt | 1 | 26.4 | 0.820295 |
| M0RAV7 | Adgrd1 | 1 | 96.1 | 0.831028 |
| Q45QK8 | Gng5 | 1 | 6.4 | 0.712739 |
| G3V6A0 | Pdgfra | 1 | 122.6 | 0.704536 |
| Q499Q2 | Tpd52l1 | 1 | 18.3 | 0.735423 |
| A0A0G2K5M1 | Sp3 | 3 | 75.4 | 0.823048 |
| P70531 | Eef2k | 2 | 81.4 | 0.741458 |
| D4A742 | Romo1 | 1 | 8.2 | 0.766194 |
| Q78E65 | Jdp2 | 1 | 18.7 | 0.749126 |
| D3Z8B9 | Fam76b | 2 | 26.6 | 0.806518 |
| Q4V8G4 | Wdr78 | 1 | 90.1 | 0.83255 |
| P0C6P7 | Fem1b | 1 | 70.2 | 0.804858 |
| P31643 | Slc6a6 | 1 | 69.8 | 0.777806 |
| Q924N5 | Acsbg1 | 1 | 80.5 | 0.745907 |
| P68943 | Med28 | 1 | 19.5 | 0.787848 |
| B0K013 | Coa4 | 1 | 10.2 | 0.795993 |
| D4A471 | Cmc2 | 1 | 9.4 | 0.787621 |
| B0BN89 | Dus2 | 1 | 55.4 | 0.570575 |
| B2RYJ5 | Tmprss13 | 1 | 59.2 | 0.784034 |
| D4A434 | Rnaseh2c | 1 | 17.9 | 0.820308 |
| D3ZDN6 | Zfp787 | 1 | 40.5 | 0.795982 |
| G3V755 | Sprr1a | 1 | 16.7 | 0.763242 |
| A0A0G2JVL6 | Ndufa8 | 2 | 20.0 | 0.830643 |
| D3ZUQ7 | Pank3 | 1 | 41.1 | 0.695227 |
| O70187 | Nfib | 3 | 47.5 | 0.800523 |
| M0RB22 | Ptprd | 1 | 217.1 | 0.771944 |
| Q9ERK1 | Camlg | 1 | 32.6 | 0.817298 |
| D4A148 | Zfr2 | 1 | 89.8 | 0.588505 |
| D3ZEK9 | Tcf15 | 1 | 20.7 | 0.759378 |
| D3ZV30 | Polr3b | 2 | 127.6 | 0.829432 |
| B3DM86 | Srxn1 | 1 | 14.2 | 0.822797 |
| A0A0G2K5M6 | Prr12 | 2 | 207.7 | 0.798383 |
| A0A0G2K7S7 | Ddr1 | 1 | 97.2 | 0.745285 |
| A0A0G2JTZ7 | Slc35f2 | 1 | 41.5 | 0.683916 |
| A0A0G2K4H8 | Ncaph2 | 1 | 71.3 | 0.761107 |
| A0A140TAE1 | Fads2 | 1 | 45.7 | 0.5907 |
